# Novel Experimental and Analysis Strategies for Fast Voltammetry: 2. A Troubleshoot-Free Flow Cell for FSCV Calibrations

**DOI:** 10.1021/acsmeasuresciau.2c00059

**Published:** 2023-01-11

**Authors:** Melissa Hexter, Joseph van Batenburg-Sherwood, Parastoo Hashemi

**Affiliations:** Department of Bioengineering, Imperial College London, SW7 2AZLondon, U.K.

**Keywords:** serotonin, flow injection analysis, 3D printing, carbon, microelectrode

## Abstract

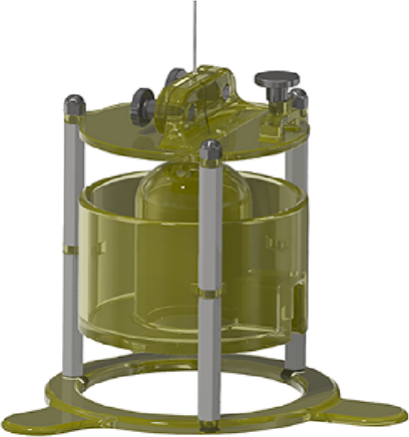

Fast scan cyclic voltammetry (FSCV) at carbon fiber microelectrodes (CFMs) is a method traditionally used for real-time quantification of neurotransmitters in biological systems. Reliable calibration of CFMs is essential for converting FSCV signals to analyte concentrations and generally employs flow injection analysis (FIA) performed with flow cells fabricated in-house. Such FSCV FIA cells often require significant and ongoing troubleshooting with pulsing, leaking, flow inconsistencies and dead volume being major causes of common challenges. In this work, we address these issues by creating a robust, plug-and-play FSCV flow cell. This novel design permits reproducible, high-precision, and stable flow injection profiles using low-cost materials to improve FSCV calibration. The ready-to-print computer-aided designs and hardware list are provided.

## Introduction

Fast scan cyclic voltammetry (FSCV) at carbon fiber micro-electrodes (CFMs) is a method traditionally used to quantify fast changes in electroactive analytes.^1^ This technique is particularly suited to monitor modulators such as dopamine, serotonin, and histamine because these molecules are of great interest biologically and have capacity for electrochemistry at fast scan rates. The community consistently develops elegant and cutting-edge solutions to refining the sensitivity and stability^2−4^ of this technique while extending the analytical scope^5−9^ to investigate physiology.

Robust calibration of the CFMs is critical for development and application of FSCV. Typically, FSCV calibration is carried out using flow injection analysis (FIA); here, the electrode is placed in a fluidic cell and a bolus of analyte is introduced to the electrode to mimic a rapid physiological event. Most flow cells are produced in-house^10,11^ and in our hands, they have suffered from irreproducibility, pulsing and other flow inconsistencies, leaking, and excess dead volume that impact the ease and reliability of calibration. Microfluidic flow cells are a viable alternative with the added benefit of on-chip dilution,^12^ but many laboratories do not have access to, nor the expertise required to employ microfabrication.

In this work, we present a macrofluidic flow cell design that can be manufactured with basic 3D printers and a number of low-cost, off-the-shelf components. The design provides pulse- and leak-free, near-square injection profiles with minimized dead volume and facilitates reproducible, low-error, stable calibrations (across different prints). Critically, the flow cell is reusable, plug-and-play, and presents minimal need for troubleshooting.

Our robust, easy-to-manufacture, and troubleshoot-free FSCV flow cell can easily be integrated into new or existing FIA systems. The ready to print computer-aided designs (CADs) are provided free of charge at www.hashemilab.com while the parts list is available in the Supporting Information (SI).

### Flow Cell Design Criteria

The motivation for this work was informed by issues with our own traditional flow injection cell designs, made in-house from glass, acrylic, or HPLC fittings.^13^

**Figure 1:**
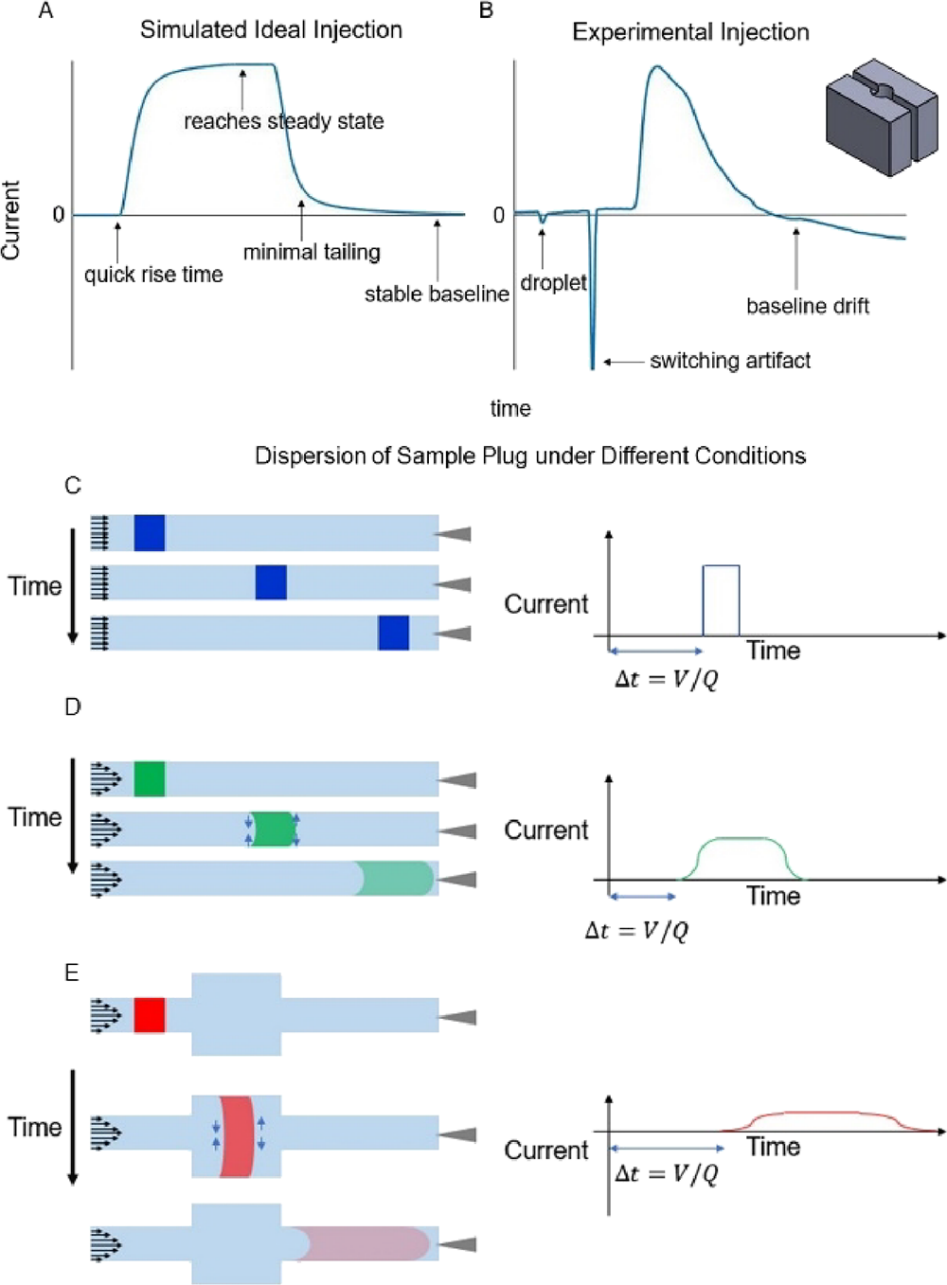
(A) Synthesized drawing of an ideal injection profile. (B) Injection profile caused by inconsistencies in the flow stream using the depicted cubic flow cell. (C–E) Evolution of the sample plug in different cases of flow characteristics and system geometry.

FSCV measures the current in response to a change in the concentration of a given analyte. The process involves background subtraction (*i.e.*, the signal of interest is the current relative to a baseline in the absence of a signal). The concentration of the analyte around the electrode can change dynamically in real systems. Therefore, FIA is ideally suited to FSCV calibrations as it provides dynamic pulses of analytes at different concentrations to the electrode. The primary function of a flow cell for FSCV hence is to robustly produce a square-like injection^14^ that indicates that the electrode has reached a steady state.^15^ A stable baseline current is required for comparison with a period of maximum, steady-state signal. After injection, the signal should rapidly return to baseline, signifying that the sample plug has passed the electrode. Any deviation from the idealized square plug at the desired concentration will result in inaccuracies. An idealized, experimental-like FSCV flow injection profile is shown in Figure 1A. Smoothing of the edges of the signal is expected experimentally because of diffusion and electrochemical kinetics. In the absence of diffusion and electrode kinetics, the sample plug would appear as it does in Figure 1C (described in detail below). Figure 1B shows an example of an injection taken with one of our previous flow cells, machined from acrylic (shown inset). The signal contains fluctuations (suggesting pulses/droplets) and artifacts (suggesting inconsistent flow and, in this case, from valve switching), does not reach steady state (due to leaking and/or dead space), and depicts a slow rise time that also indicates the presence of dead space. Such systems typically require significant and consistent (*i.e.*, before each experiment) troubleshooting to get responses close to ideal. We therefore set strict design criteria for an optimized flow cell response. These criteria were to reduce flow inconsistencies (a), leaks (b), and dead volume (c) (see design features). Flow inconsistencies and leaks are intuitive issues, but dead volume is dually problematic because it permits dispersion of the sample plug (reducing the peak concentration) while also increasing time of detection. The distortion of the injection profile due to dispersion, exacerbated by dead space, is highlighted in Figure 1E. This distortion can increase the signal duration beyond the acquisition window of FSCV, emphasizing the importance of mitigating dead space.

Figure 1C–E depicts the evolution of a sample plug over time under different fluid characteristics and system parameters to highlight the role of dispersion in the flow injection analysis system. The blue sample plug in Figure 1C corresponds to the progression of an inviscid fluid with uniform velocity leading to no dispersion. The green sample plug in Figure 1D demonstrates a fluid with a given viscosity under the no-slip condition, prompting a parabolic flow profile. With a parabolic flow profile, the solute in the center moves faster than at the edges, generating radial concentration gradients that drive Taylor dispersion. The red sample plug in Figure 1E has the same flow characteristics as the green sample plug with added dead volume. This additional dead space increases the time of delivery to the electrode and reshapes the bolus as a result of additional dispersion.

In sum, our designs (described in detail throughout the rest of the paper) address flow inconsistencies, leaks, and dead volume, which are the primary culprits of irreproducible FSCV calibrations.

## Experimental Section

### Fluid Analysis

The flow characteristics of the cycling well in the flow cell were predicted using eq 1, which allowed us to calculate the Reynolds number (Re) in a cylinder. The Reynolds number is the ratio of inertial forces to viscous forces where *Q* is the flow rate, ρ is the density of the solution, μ is the viscosity of the solution, and *d* is the diameter of the cycling well (3 mm). In this analysis, we assume the viscosity to be that of water, 1 mPa·s, given the low concentration of solutes in the solutions used. We calculated the Reynolds number of the flow entering the well to be ∼0.0025, and hence the flow is laminar.
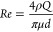
1The transport characteristics of the analyte to the electrode surface were described using eq 2. We determined the Peclet number (Pe) by considering the diffusion coefficient of serotonin (*D*_ser_) as reported in the literature^16^ (*D*_ser_ = 5.4 × 10^–6^ cm^2^ s^–1^) and the velocity of the fluid (*v*). The velocity of the fluid was calculated by dividing the flow rate of 1.7 mL min^−1^by the cross-sectional area of the tubing. The Peclet number determines the ratio of advective to diffusive transport in a system. The delivery of the analyte to the electrode is governed by advection, based on the estimated Peclet Number, 1400.
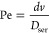
2

### Flow Cell Fabrication and Design

The flow cell was designed using Inventor (Autodesk, San Francisco, CA, USA) and 3D printed. To evaluate whether the 3D printer selected affected the performance, we made devices using both polyjet (Objet Pro with Veroclear resin, Stratsys, Gothenburg, Sweden) and stereolithographic (Formlabs Form 3 with clear resin, MA, USA) printers. The stereolithography-printed products were washed in a 99% isopropyl alcohol (Sigma-Aldrich, MO, USA) bath and UV-cured for 10 min. Following the print phase, a nylon 1/4-28 UNF nut was secured to the inlet of the flow cell using an epoxy. After curing the epoxy overnight, an HPLC fitting was screwed into the nut to create a watertight seal. The flow cell was assembled using stainless-steel standoffs, M3 dome nuts, M3 thumb screws, M3 hexagon nuts, and M3 countersunk screws. The inlet for the thumb screw on the head stage mount was hand-tapped to create 3 mm threads.

### Flow Injection Analysis

**Figure 2:**
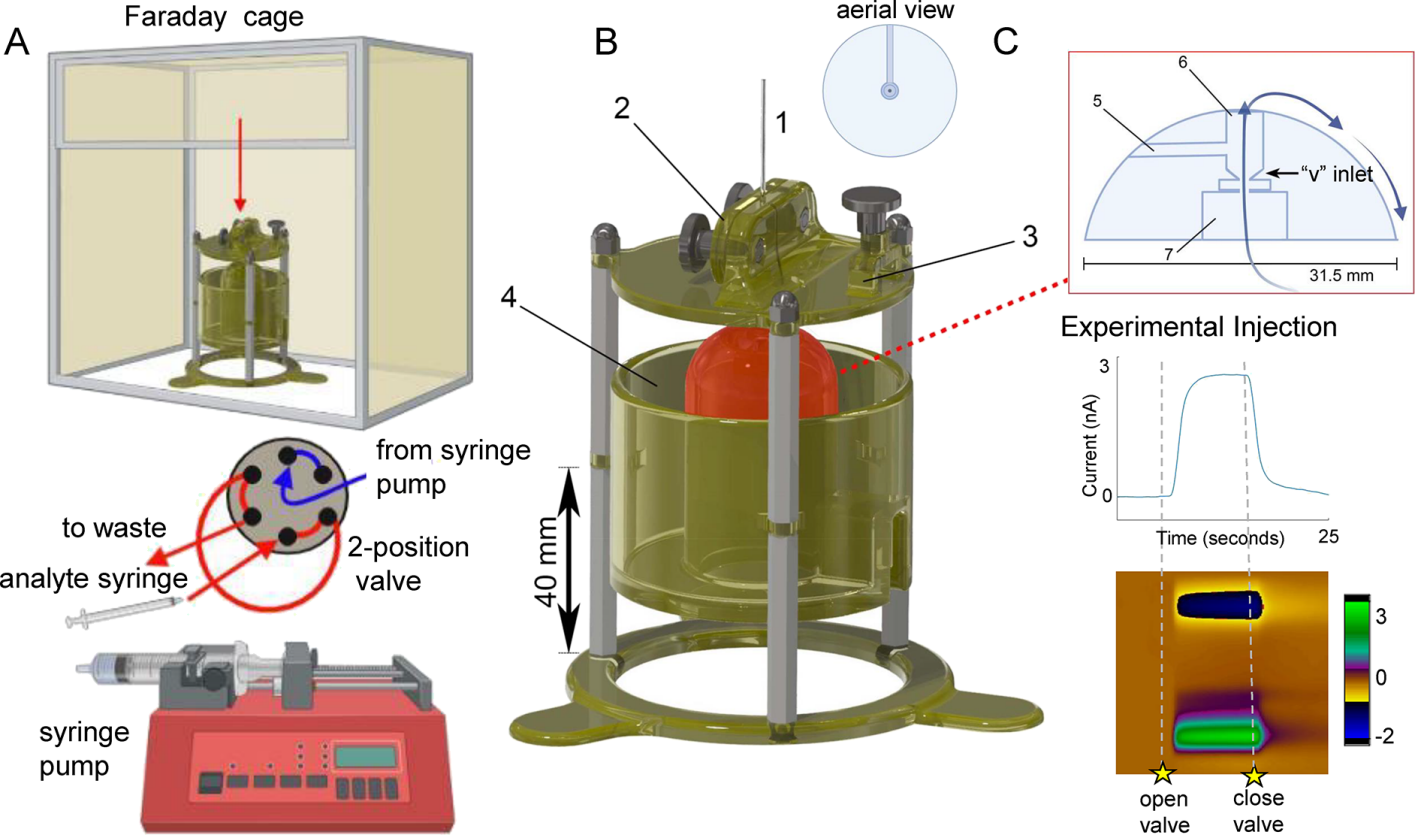
(A) Flow injection apparatus with a flow cell, Faraday cage, six-port two-position valve, syringe pump, and sample syringe. (B) Design of a novel flow cell with additional lateral view of dome internals. (C) Current versus time plot (above) and color plot with the current in false color (below) of experimental injection of 100 nM serotonin in a flow cell that achieves a steady state at the peak current (in nA) with minimal tailing and no baseline artifacts.

The flow injection analysis system consisted of a syringe pump (Harvard Apparatus, MA, USA), six-port HPLC valve (VICI, Valco, Houston, TX, USA) and flow cell (Figure 2A). The flow rate was optimized to 1.7 mL min^−1^ to minimize the reagent used and rise time of the sample plug while reducing tailing. This system operated best with flow rates in the range of 1–2 mL min^-1^. The HPLC valve was left in the inject position for 10 s during sample injections of 1 mL from a 5 mL syringe of calibration standard. The ideal sample loop tubing was 22 in., the inlet tubing length was 6 in., and the tubing inner diameter was 0.04 in. (PEEK). This sample loop size guaranteed that the sample plug was long enough to reach a steady state. Note that these parameters will likely change with different tubing sizes.

### Solutions

Stock solutions of serotonin HCL and l-ascorbic acid (Sigma-Aldrich, MO) were prepared in a physiological salt buffer (15 mM Tris, 126 mM NaCl, 2.5 mM KCl, 25 mM NaHCO_3_, 2.4 mM CaCl_2_, 1.2 mM NaH_2_PO_4_, 1.2 mM MgCl_2_, 2.0 mM Na_2_SO_4_) maintained at a pH of 7.4. L-Glutamic acid was added to the buffer solution during electrode cycling to achieve a concentration of 1 μM.

### Electrode Fabrication

Cylindrical CFMs were fabricated by hand as previously described.^17^ Carbon fibers were trimmed to 150 μm under a stereoscope before being electrocoated with Nafion (Liquion-1105-MeOH, Ion Power, DE, USA) as described previously.^18^ Pseudo-reference electrodes were created by electroplating silver wire (A-M systems, WA, USA) with chloride ions at 5 V for 30 s in a solution of 0.1 M HCl.

### FSCV

FSCV was performed using a Pine Research head stage (Pine Research Instrumentation, Durham, NC, USA) connected to a potentiostat (Dagan Corporation, Minneapolis, MN, USA). The potentiostat was controlled by the WCCV 3.06 software (Knowmad Technologies LLC, Tucson, AZ, United States) *via* a USB-6431 DAC/ADC (National Instruments, TX, USA) device. For pre-treatment of the electrode, a modified triangular waveform scanning from 0.2 to −0.1 to 1.3 to 0.2 V at a rate of 1000 V/was applied at 60 Hz for 10 min and then 10 Hz for 10 min in the salts buffer with 1 μM l-glutamic acid. The waveform was then switched to the Jackson waveform^19^ and cycled at 60 Hz for 10 min and then 10 Hz for 10 min for a total of 40 min of cycling. Data was smoothed and treated using a Butterworth filter and a 5 kHz low-pass filter.

## Results/Discussion

Our new flow cell design is depicted in Figure 2. The flow injection apparatus includes a syringe pump, a six-port two-position valve, the flow cell inside a Faraday cage (not to scale), and a syringe containing a sample. The red arrow above the flow cell in the Faraday cage in Figure 2A depicts where the electrode is placed into the flow cell. The syringe pump delivers FIA buffer, and the analyte syringe transports the sample to the valve. The valve introduces a known volume of sample into the flow cell that is observed as a rapid pulse by the electrode (1) in Figure 2B)).

Figure 2B shows a rendering of the flow cell design. The electrode mount (2) accurately and reproducibly centers the electrode in the cycling well in order to guarantee that the electrode is consistently exposed to the maximum concentration of solute in the sample plug. Previous work has highlighted the importance of the position of the microelectrode in the flow stream due to dispersion.^10^ The mount also eliminates the need for an expensive micromanipulator. The head stage mount (3) secures the head stage to prevent damage to the electrode *via* mechanical stress on the wires connecting the head stage to the acquisition system. After passing the electrode, the fluid exits the cycling well, cascading over the top of the dome-shaped flow cell into a waste reservoir (4). The design has a shallow channel along the entirety of the dome to prevent snaking and ensure a more stable flow to the waste. This shallow channel can be seen in the aerial view of the flow cell depicted in Figure 2B. The flow cell includes a channel for the reference electrode (5), a flow stream from the valve that enters the underside of the cycling well (6), the area for the hex nut (7) to fasten the inlet fitting, and the V-shaped inlet to the cycling well.

### Design Features

Based on the determined flow characteristics of this system (stated in the Experimental Section), it is therefore not surprising that pulsing and flow inconsistencies (a), leaks (b), and dead volume (c) predominantly impact the profile and quality of the sample injection *via* perturbation of the flow stream.

(a). To mitigate pulses and flow inconsistencies, we designed a dome shape to encompass the flow cell outlet. This configuration eliminates pulses in the flow stream because curved surfaces minimize chaotic variation in fluid velocity and therefore improve the reproducibility of injections. The curved design permits an infinitesimal change in fluid velocity down the curvature of the dome to maintain pulse-free, laminar flow. The use of a reservoir also minimizes pulsing in the flow stream that is observed when using other waste management strategies. For example, droplet formation occurs when using conventional tubing as an outlet for this system. The reservoir volume is approximately 60 mL, meaning that waste fluid can be removed relatively infrequently during experiments. Finally, the lack of outlet tubing, enabled by this open fluidic design, manages the pressure change to minimize flow inconsistencies caused by valve switching. This design configuration permits rapid equilibration with atmospheric pressure in contrast to a closed fluidic system, where the pressure differential can only be remedied after it reaches the end of the outlet tubing.
(b). To minimize leaking, the 3D printing methods (polyjet and stereolithography) were chosen for their resolution and material compatibility. These high-resolution printing techniques create water resistance to eliminate leaks through the structures of the flow cell. The resin materials were chosen based on color and printer compatibility. The selection of a clear resin permitted easy visualization of the flow stream and flow cell channels. Additionally, high-pressure male fittings and their female hex nut counterparts were appropriately selected to ensure good seals. The depth of the underside indentation in the flow cell was chosen to match the size of the selected fittings to create a watertight seal.
(c). Dead volume detracts from the ideal injection by permitting dispersion of the sample plug and increasing analyte detection time (Figure 1C). Increasing the time of analyte detection is a critical concern for a technique that is characterized by superior temporal resolution. We engineered a tapered inlet (labeled v inlet on Figure 2C) to focus the flow stream to minimize dispersion of the sample during transport. This inlet shape also reduces the total internal volume of the channel in the flow cell to minimize dead space. This reduced internal volume permits a slower flow rate while maintaining the same time of detection. A decrease in flow rate enables the reduction of buffer per injection, thereby lowering the cost of experiments. The space for the hex nut fitting was minimized to ensure proximity between the end of the tubing from the HPLC valve to the tip of the electrode. This approach reduced dead space and improved design reproducibility by removing the need for manual tapping to create threads for the inlet fitting. Finally, we used a narrower 3 mm diameter cycling well for optimized well turnover time.

With this optimized design, we found square-like analyte injections with an example illustrated in Figure 2C. The signal rises and falls with minimal tailing and reaches a steady state within the time collection window. In the next sections, we will address reproducibility and stability of the flow cell.

### Robustness and Reproducibility

**Figure 3:**
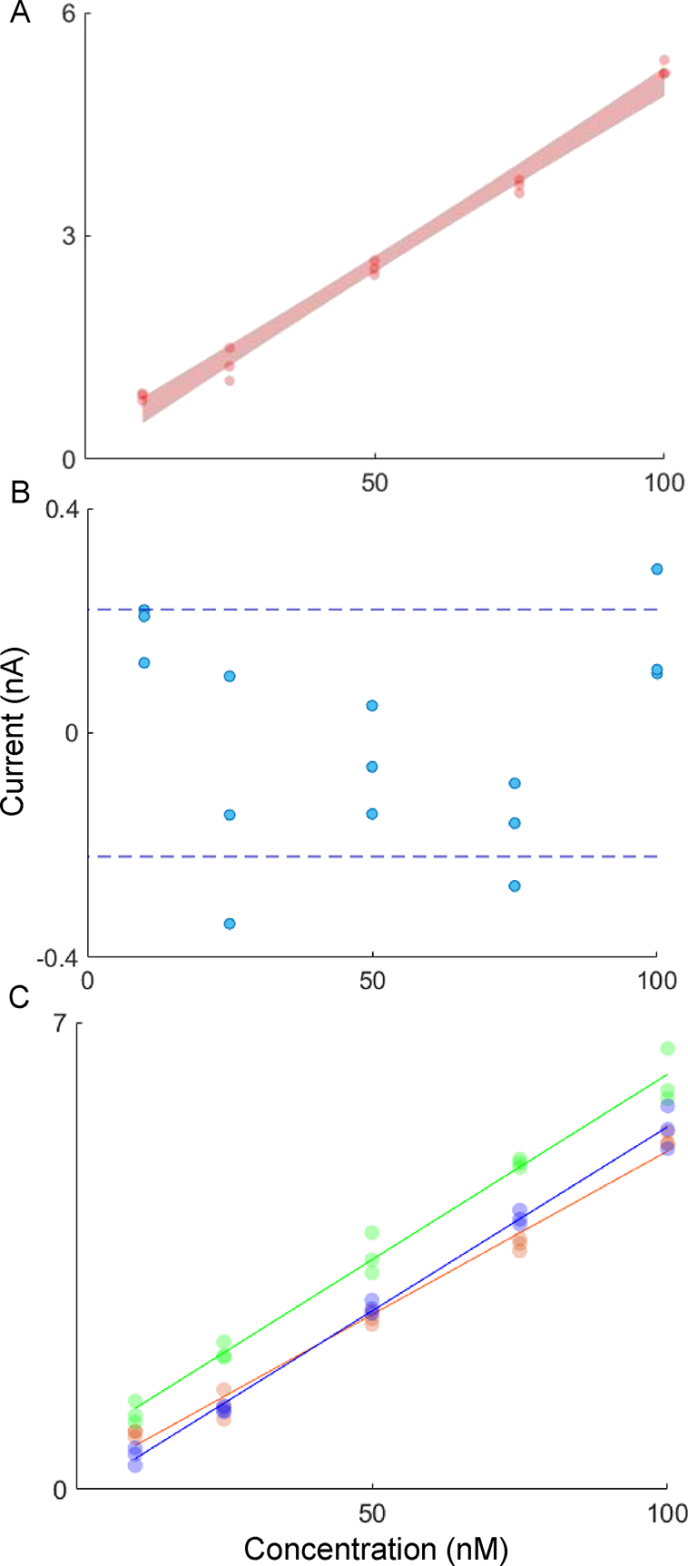
Measurements of serotonin at various concentrations. (A) Representative calibration curve with shaded 95% confidence bands. (B) Residuals of the calibration with uncertainty as the standard deviation of the residuals indicated by dashed lines. (C) Calibration curves for serotonin measurement with (*n* = 3) different Nafion electrodeposited electrodes in one flow cell. Electrodes were pretreated with glutamate as stated in the Experimental Section.

To evaluate robustness and reproducibility within a print, we first performed five-point FSCV calibrations with serotonin concentrations of 10, 25, 50, 75, and 100 nM on one electrode. In this concentration range, we can estimate the concentration of serotonin with an uncertainty of 0.2 nM. In Figure 3A, the resulting calibration curve (three injections averaged per concentration) illustrates the spread of the data with the 95% confidence bands shaded in red.

Figure 3B depicts the residuals of all collected data for each calibration point from Figure 3A. The residuals are mostly randomly distributed, suggesting that linear regression is an appropriate fit for the data. The minimum and maximum concentration values have positive values, but this is likely due to the order of measurement given that electrode sensitivity decays with consecutive injections of serotonin. There are nine calibrations in this data set, so there may be small sample bias with a random generator to determine the order of calibration standards introduced. Previous work also implies the suitability of a linear regression for this concentration range;^18^ however, it is important to note that the solution matrix can substantially alter the slope of calibrations (see SI Figure S1 for a comparison of calibration in glutamate versus glutamate, gamma-aminobutyric acid, and glycine in physiological buffer). Despite matrix effects, it is essential to perform calibration to approximate the levels of relevant analytes for biomarker development and the elucidation of physiochemical kinetics.

Next, we performed five-point FSCV calibrations with three separate electrodes. Figure 3C shows that these calibrations are highly reproducible. In fact, no significant difference between the residuals for each concentration point of each electrode (*p* > 0.05) was found when the data was treated with two-way ANOVA. The uncertainty of estimate (UOE) is 0.2 nM for both the representative calibration and the mean UOE for all calibration curves performed in a single flow cell. These parameters attest to the robustness of individual injections for each calibration point even though electrodes lose sensitivity with time *in vitro* after serotonin injections.^20^

Next, we probed reproducibility between prints on the same printer. Here, two flow cells were printed from the same polyjet printer with the same material to examine the effect of print variability within the same printer on reproducibility. Calibrations in these two cells were compared to a third print using a different printer, material, and printing method.

**Figure 4:**
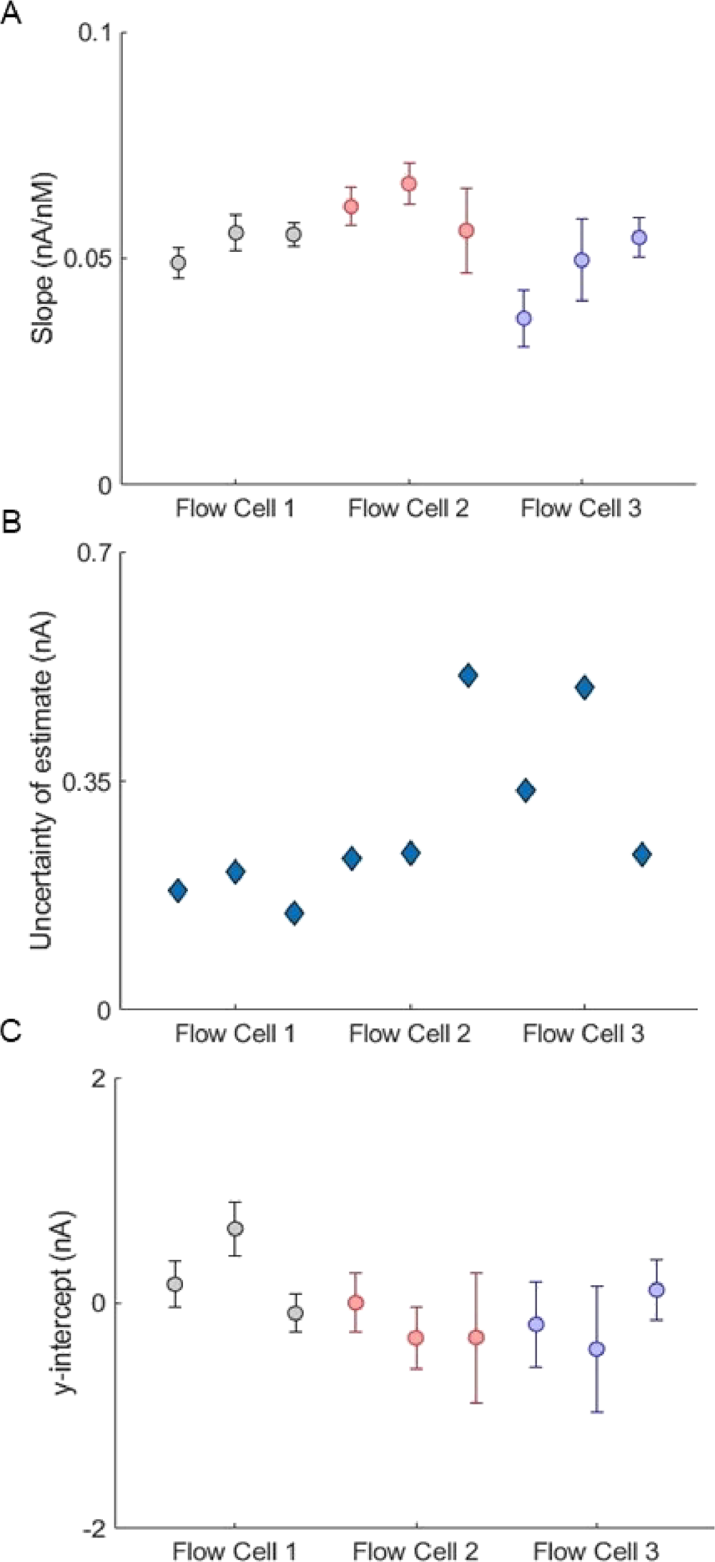
Evaluation of 95% confidence interval for given regression parameters and uncertainty of estimate for each flow cell print. (A) Slope for each linear regression with 95% confidence interval bounds. (B) Standard deviation of the regression for each calibration curve. (C) *y* intercept for each linear regression.

An important point of note is that each print, once constructed with appropriate hardware, was instantly functional and did not require any troubleshooting to obtain these calibrations. To test if there was any significant variation in our calibrations across prints, the 95% confidence interval of the slope, the UOE, and 95% confidence interval of the intercept were examined in Figure 4A–C, respectively. There was no significant variation between calibration parameters obtained across prints as determined by ANOVA (all *p* > 0.05), indicating that print variability within the same or a different printer, material, and printing method does not affect the performance of the flow cell. Given the highly reproducible nature of our design, we next probed the short and long stability of the flow injections.

### Stability

Stability of the flow injections over time are a critical consideration for *in vitro* work. Here, we probed both short (hours) and long (days) term stability of the reproducibility of our injections with our novel design.

**Figure 5:**
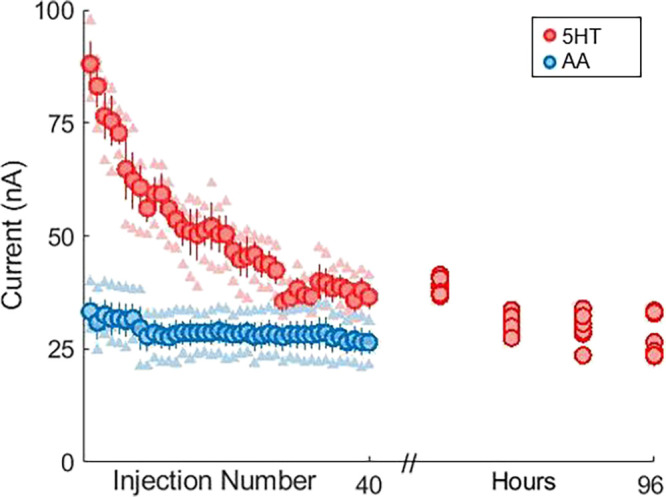
Multiple trials of 40 successive injections of 1 μM serotonin (red) and 100 μM ascorbic acid (blue) with corresponding current values followed by a single injection of 1 μM serotonin every 24 h for 96 h. (5-HT = serotonin AA = ascorbic acid).

We started by injecting serotonin (1 μM) onto three separate electrodes every 2 min, and the average of these measurements is shown in Figure 5 (red markers). After 40 injections (lasting 80 min), the flow was turned off, the flow cell was washed, and the electrodes were stored. The same electrodes were employed once every 24 h (with four injections of fresh solutions) for the next 4 days. There is considerable loss of signal over the first 25 injections that is depicted in Figure 5. The loss in current becomes stable after 40 ± 2 injections based on the time constant derived from an exponential fitting of the decay (*y* = 78.25e^–0.0201*x*^) performed in Matlab. This fitting also accurately predicts the loss in current over the consecutive 4 days. This loss is not due to the flow cell because the coefficient of variation of the first 10 injections is not statistically different (*p* ≫ 0.05 by *t*-test) from that of the last 10 of the injections over 96 h. Additionally, we have observed this behavior previously with serotonin injections *in vitro* (but not *in vivo*).^20^

In a previous study,^20^ we attributed the loss in signal to polymerization of serotonin and its metabolites on the electrode surface (a phenomenon that is mitigated *in vivo* due to a complex interaction of other ambient amino acids polymerizing on the electrode). Here, we confirmed that this effect was due to the chemistry of serotonin with the electrode and not due to stability issues with the flow cell by performing a second set of experiments. In these experiments, the chosen analyte is ascorbic acid (AA), which does not have the same potential for electropolymerization due to the lack of a terminal amine group. It is clear from Figure 5 that there is minimal change in the ascorbic acid signal over time. These experiments attest to the high level of stability of injections performed with our new design and furthermore that the decay in the current is uniquely an electrochemical phenomenon.

## Conclusions

Calibration of CFMs for FSCV analysis of neurotransmitters has been challenging due to extensive troubleshooting of in-house-developed flow cells. These flow cells were plagued by pulsing, leaks, flow inconsistencies, and large dead space that together yielded unreliable flow injection profiles. In this work, we minimized these problems by considering the fluid dynamics in a novel flow cell design. Key design features included the v inlet, dome-shaped outlet, electrode mount, and waste reservoir. This flow cell generated reproducible, low-error, and stable flow injection profiles despite variation caused by hand-fabrication of electrodes and complex electrochemical phenomena. The CADs and parts list are provided (www.hashemilab.com) and can be printed with low-cost materials. Calibrations could be further improved with additional work on other aspects of the FIA system, such as the six-port HPLC valve, which has a switching mechanism that can cause a pressure differential detectable by the electrode.

## Supporting Information Available

Calibration curves in differing matrices with calculated error and parts list for flow cell design (PDF)

## Abbreviations

FSCV: fast-scan cyclic voltammetry;
5HT: 5-hydroxytryptamine;
