# Supplementary material for: Novel Experimental and Analysis Strategies for Fast Voltammetry: 2. A Troubleshoot-Free Flow Cell for FSCV Calibrations
Source: ACS Meas Sci Au. 2023 Jan 11;3(2):120–6. doi: 10.1021/acsmeasuresciau.2c00059 (PMC10120031; doi:10.1021/acsmeasuresciau.2c00059)
Supplement: Supplementary file 1 — tg2c00059_si_001.pdf [file tg2c00059_si_001.pdf]

Supporting information for

Novel Experimental and Analysis Strategies for Fast Voltammetry: 3. A Troubleshoot-Free Flow Cell for FSCV Calibrations

Melissa Hexter[1], Joseph van Batenburg-Sherwood[1] and Parastoo Hashemi[1*]

[1] Department of Bioengineering, Imperial College London, SW7 2AZ UK

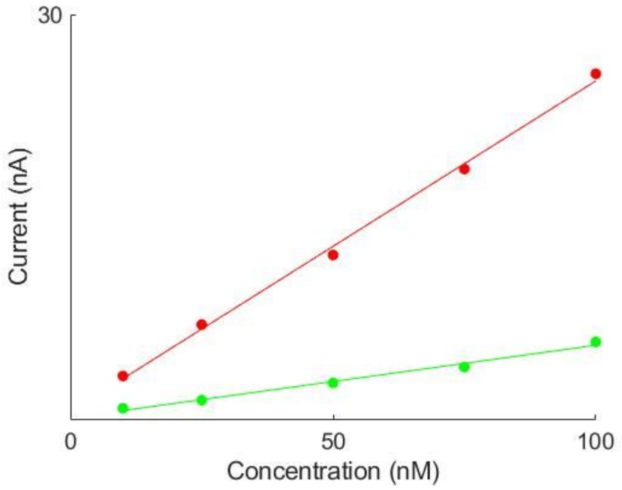

Figure S1 Calibration curve performed in glutamate only (red) versus glutamate, gamma-aminobutyric acid (GABA), and glycine (green).

The prediction error is low when concentrations are estimated from calibration curves from the same matrix. In contrast, the prediction error is extraordinarily high when the estimated concentrations are from a different matrix from that of the calibration curve. The extraordinary difference (calculated via root mean square error (RMSE)) between the estimated and true values for concentrations in differing matrices can be seen in **Table S1.**

Table S1

matrix	RMSE (nA)
same	0.2877
different	226

Parts List for Flow Cell:

part	quantity (per flow cell)	supplier
Stainless steel standoff, Male-Female, 40 mm, 46 mm	6	Farnell
M3 dome nuts	3	Accu
M3 x 8mm thumb screws	3	Accu
M3 x 6mm countersunk screw	3	Accu
¼-28 hex nut	1	Zoro
